# Adsorption of extracellular proteases and pyocyanin produced by *Pseudomonas aeruginosa* using a macroporous magnesium oxide-templated carbon decreases cytotoxicity

**DOI:** 10.1016/j.crmicr.2022.100160

**Published:** 2022-08-18

**Authors:** Hidetada Hirakawa, Ayuko Kimura, Ayako Takita, Sayaka Chihara, Koichi Tanimoto, Haruyoshi Tomita

**Affiliations:** a Department of Bacteriology, Gunma University, Graduate School of Medicine, 3-39-22 Showa-machi Maebashi, Gunma 371-8511, Japan; b Graduate School of Health Science, Gunma Paz University, Tonyamachi 1-7-1, Takasaki City, Gunma 370-0006, Japan; c Laboratory of Bacterial Drug Resistance, Gunma University, Graduate School of Medicine, 3-39-22 Showa-machi Maebashi, Gunma 371-8511

**Keywords:** Antimicrobial resistance (AMR), Virulence, Extracellular protease, Porous carbon, Adsorbent, Bacterial translocation

## Abstract

*Pseudomonas aeruginosa* is one of the most common pathogens isolated in clinical settings and produces a wide range of extracellular molecules that contributes to the virulence. Chemotherapy options to prevent and treat *P. aeruginosa* infections are limited because this pathogen is highly and innately resistant to some classes of conventional drugs. Alternative methods to conquer *P. aeruginosa*, including multidrug resistant strains, are being investigated. This study showed that a macroporous magnesium oxide (MgO)-templated carbon material (MgOC_150_) attenuates the toxicity of this bacterium in human epithelial cells. A proteomic analysis revealed that MgOC_150_ adsorbs some extracellular proteases, including elastase (LasB) and alkaline protease (AprA), required for the virulence of *P. aeruginosa*, which decreases the accumulation of these molecules. MgOC_150_ also adsorbed pyocyanin, which is another molecule involved in its pathogenesis, but is a nonprotein small-sized molecule. These results suggest a potency of MgOC_150_ that suppresses the virulence of *P. aeruginosa*. MgOC_150_ has been used for industrial purposes, as an electrode catalyst and a bioelectrode and for enzyme immobilization. Thus, MgOC_150_ could be beneficial for developing novel anti-Pseudomonas therapy.

## Highlights

• Cytotoxicity of *P. aeruginosa* was attenuated by the macroporous adsorbent MgOC_150_.
• MgOC_150_ did not disturb human epithelial cells.
• MgOC_150_ decreased extracellular proteolytic activity and pyocyanin level.
• MgOC_150_ effectively adsorbed elastase, alkaline protease and the pyocyanin molecule.
• The MgOC_150_ adsorbent is beneficial for developing anti-Pseudomonas therapy.

## Graphical Abstract

**Figure:**
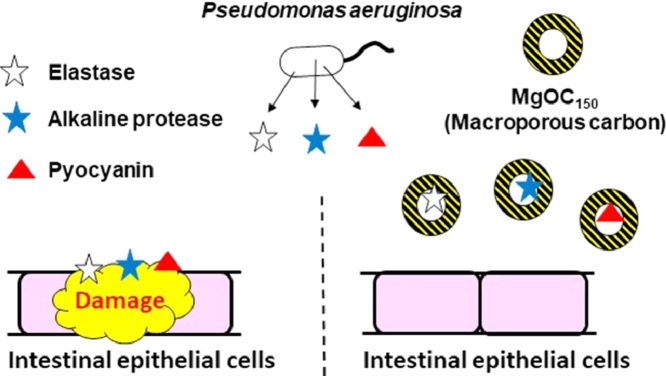

## 1 Introduction

*Pseudomonas aeruginosa* is a clinically important pathogen. This bacterium is frequently isolated with nosocomial infections, and it causes serious infections in immunocompromised individuals and patients who have chronic respiratory diseases, such as cystic fibrosis (CF) and diffuse panbronchiolitis (Murray et al., 2007, Poletti et al., 2006). Although a major site of *P. aeruginosa* infection is the lungs, a considerable number of fatal systemic infections occur when it becomes bacteremic (Turner et al., 2014, Mittal et al., 2009, Zaborina et al., 2006). This bacterium is not a common intestinal pathogen in healthy hosts however, *P. aeruginosa* colonizes in the gut of hospitalized, immunocompromised, antibiotic-treated patients or patients with cancer, and it disrupts the intestinal epithelial barrier, leading to bloodstream infections (Adlard et al., 1998, Gomez-Zorrilla et al., 2015, Balzan et al., 2007, Okuda et al., 2010, Markou and Apidianakis, 2014). *P. aeruginosa* infection is generally difficult to treat because it is innately resistant to many classes of antimicrobial agents and there are multidrug-resistant strains (Poole, 2011, Hoiby, 2011, Aloush et al., 2006, Obritsch et al., 2004). For these reasons, developing any strategies to prevent and/or ameliorate infections caused by this pathogen is necessary.

Porous carbon can adsorb certain organic molecules that fit its internal pore. Activated charcoal is the most well-known substrate of porous carbons. It is used to remove toxic substances in industrial applications while several activated charcoal materials can be used for medical purposes as oral medicines, such as treating intoxications and certain circulatory diseases by adsorbing body wastes (Hegade et al., 2015, Scaldaferri et al., 2013, Zellner et al., 2019). For instance, AST120 (Kremezin) is used to treat progressive chronic kidney disease by adsorbing and removing uremic toxin precursors produced by gut microorganisms (Niwa, 2011). Pyocyanin is the most-characterized non-protein molecule that contributes to virulence of *P. aeruginosa*, and it impairs host cells by generating reactive oxygen species (Hall et al., 2016). We previously found that AST-120, adsorbs pyocyanin produced by *P. aeruginosa,* and attenuated pyocyanin-associated toxicity to intestinal epithelial cells (Hirakawa et al., 2021b). This finding leads to the focus of potency of porous carbon as an anti-Pseudomonas option. The average pore size of AST-120 is ∼2 nm, and this pore size is suitable for the high adsorption of the pyocyanin molecule. However, it is presumably too small to adsorb relatively large-sized protein molecules. Virulence of *P. aeruginosa* is supported by not only certain non-protein molecules, including pyocyanin, but also various extracellular protein molecules, such as elastase and alkaline protease (Gellatly and Hancock, 2013, Hauser, 2009, Jenkins et al., 2004, Kipnis et al., 2006, O'Callaghan et al., 2019).

Magnesium oxide (MgO)-templated carbon (MgOC) is another recently industrialized porous carbon. Its pore producing process is different from activated charcoal (Inagaki et al., 2004). In contrast, pores of activated charcoal are generated in the activation process. Pores of MgOC are produced by a distinct method from conventional activation. The pore template is formed by incorporating the MgO molecule generated during the pyrolysis of an Mg-containing organic substrate, such as magnesium citrate and magnesium gluconate, into a carbon matrix, and the pore is produced by removing the MgO molecule (Morishita et al., 2010). The pore size of the resulting MgOC can be precisely predesigned according to this method. This study aims to develop a strategy that suppresses the virulence of intestinal *P. aeruginosa*. It relies on adsorbing extracellular molecules required for bacterial virulence. MgOC material with an average pore size of 150 nm, named MgOC_150_, was utilized because this size is predicted to adsorb protein molecules >30 kDa (Funabashi et al., 2017). This study demonstrated that the MgOC material could adsorb extracellular proteases and pyocyanin produced by *P. aeruginosa,* including clinical isolates that are resistant to antimicrobials, such as multidrug-resistant *P. aeruginosa* (MDRP), and attenuate the toxicity to host intestinal epithelial cells.

## 2 Materials and Methods

### 2.1 Bacterial strains, host cells, and culture conditions

We used *P. aeruginosa* PAO1 strain and several non-PAO1 antibiotic-resistant clinical isolates (including the MDRP strains) as listed in Table 1. Ps.a-885, Ps.a-890, Ps.a-1016 and RPs.a-884 were isolated from the feces. Ps.a-1200, Ps.a-1205 and RPs.a-914 were isolated from the sputum while RPs.a-946 was isolated from the urine. The bacteria were aerobically grown at 37°C. Caco-2 (ATCC HTB-37) cells were obtained from American Type Culture Collection (ATCC) and cultured in Dulbecco's modified Eagle medium (DMEM) containing 10% HyClone FetalClone III serum (HyClone Laboratories, Inc., Logan, UT, USA) at 37°C, 5% CO_2_.

**Table 1:** Drug-resistant clinical isolates used in this study.

Strains	Specimen	Resistance to:
Ps.a-885	Feces	Levofloxacin
Ps.a 890	Feces	Ceftazidime
Ps.a-1016	Feces	Imipenem
Ps.a-1200	Sputum	Levofloxacin
Ps.a-1205	Sputum	Aztreonam, Meropenem, Levofloxacin
RPs.a-884	Feces	Ceftazidime, Imipenem, Meropenem, Cefepime, Gentamycin,Amikacin, Levofloxacin
RPs.a-914	Sputum	Imipenem, Meropenem, Gentamycin, Amikacin, Levofloxacin
RPs.a-946	Urine	Imipenem, Meropenem, Gentamycin, Amikacin, Levofloxacin

### 2.2 Cytotoxicity assay

Cytotoxicity of bacterial cultures to human intestinal epithelial cells was estimated in a cell viability assay as previously described (Hirakawa et al., 2021b). Bacteria were grown in LB medium for 24 h. A total of the 50 μL of bacteria-free culture supernatants was diluted into 50 μL DMEM containing 10% HyClone FetalClone III serum, and added to cultured Caco-2 cells in a 96-well plate. As a control, twofold diluted LB medium in DMEM containing 10% HyClone FetalClone III serum was added to the host cells. After incubation for 24 h, the cell viabilities were determined by measuring intracellular ATP levels with the CellTiter-Glo Luminescent Cell Viability Assay (Promega Corp., Madison, WI, USA). The cell viabilities were represented as relative light units (RLUs) by their ratios (%) to the RLU of the control sample. To estimate cytotoxic effects of MgOC_150_ to human intestinal epithelial cells, Caco-2 cells were incubated with MgOC_150_ in 150 μL DMEM containing 10% HyClone FetalClone III serum for 24 h and the cell viabilities were determined.

### 2.3 Protease assay

Protease activity was determined by measuring a proteolytic digestion of azocasein, a protease substrate. To measure the extracellular protease level in a bacterial culture, bacteria were grown in LB medium for 24 h. In addition, 7.5 μL of bacteria-free culture supernatant was added into a 50 mM phosphate buffer (pH 7.5) containing 1 mg azocasein. To determine protease activity for purified recombinant proteins, 5 μg of each protein was incubated with 1 mg azocasein in a 50 mM phosphate buffer (pH 7.5) containing 1 mM CaCl_2_ and 0.1 mM ZnCl_2_. After incubation for 1 h at 37°C, 3 % trichloroacetic acid (TCA) was added, and the protease activity was quantified by measuring the absorbance at 366 nm.

### 2.4 SDS-polyacrylamide gel electrophoresis (SDS-PAGE) and mass spectrometry analyses

The PAO1 strains were grown with and without 30 mg MgOC_150_ in 5 mL of LB medium for 8 h, and separated by centrifugation and filtration. Extracellular proteins were precipitated from 1350 μL supernatants with 10% trichloroacetic acid (TCA) and dissolved in 60 μL of the Laemmli sample buffer (Bio-Rad Laboratories, Hercules, CA, USA). A total of 30 μL of the protein solutions were separated by SDS-PAGE with a 10% gel and stained with Coomassie brilliant blue (CBB). Two protein bands, corresponding to approximately 35 and 20 kDa, respectively, were excised and subjected to in-gel digestion using trypsin (Promega Corp., Madison, WI, USA) at 37 ˚C overnight. Peptides were desalted with C18 StageTips. LC-MS/MS analysis was performed using a Dionex Ultimate 3000 nano HPLC system (Thermo Fisher Scientific, Bremen, Germany) coupled to a LTQ Orbitrap Velos (Thermo Fisher Scientific). Before injection into the mass spectrometer, peptides were loaded online in a trap column Acclaim™ PepMap™ 100 C18 LC column (5 μm particle size, 100Å pore size, 0.1 mm×20 mm; Thermo Fisher Scientific) and separated by a nanoscale C18 capillary LC column (NTCC-360/75-3-125; Nikkyo Technos, Tokyo, Japan) by a linear gradient of acetonitrile (4-33%) in 0.1% formic acid for 120 min at a flow rate of 0.3μL/min and directly electrosprayed with electrospray ionization (ESI) source in a positive ion mode. The mass spectrometric conditions were as follows: m/z range, 350–1500; spray voltage, 2.5 kV; capillary temperature, 250°C; normalized collision energy, 35.0%; isolation width, 1 m/z; activation time, 10 ms; activation Q,0.25; dynamic exclusion, 60 s; resolution, 30,000; and data dependent mode with product ion scans for the 15 most intense ions in the full-scan mass spectrum. Peptide identification and label-free quantitation (LFQ) were performed using MaxQuant software (ver.1.6.2.10, Cox and Mann, 2008) using default settings (1% overall peptide false discovery rate) with slight modifications as follows: main search peptide tolerance, 6 ppm; and variable modifications, Oxidation (M), Acetylation (Protein N-term), and Carbamidomethylation (C). The proteomics data have been deposited to the ProteomeXchange Consortium via the jPOST (Moriya et al., 2019) with dataset identifier PXD032870.

### 2.5 Plasmid construction

To construct IPTG-inducible C-terminally histidine-tagged LasB, AprA, PA0423 and LolA expression plasmids pET28a-lasB-His, pET28a-aprA-His, pET42c-PA0423-His and pET42c-lolA-His, respectively, we PCR-amplified the *lasB, aprA, PA0423* and *lolA* genes with the primer pairs listed in Table 2. These PCR products were digested with NcoI and HindIII for the *lasB* and *aprA* genes and NdeI and XhoI for the *PA0423* and *lolA* genes, and ligated into pET28a (for the *lasB* and *aprA* genes) and pET42c (for the *PA0423* and *lolA* genes) plasmids. All constructs were confirmed by DNA sequencing.

**Table 2:** Primers used in this study.

Primer	DNA sequence (5’ – 3’)	Use
pET lasB F	gcgccatggagaaggtttctacgcttg	pET28a-lasB-His construction
pET-lasB-R	gcgaagcttcaacgcgctcgggcaggtc	pET28a-lasB-His construction
pET-aprA-F	gcgccatggccagcaattctcttgc	pET28a-aprA-His construction
pET-aprA-R	gcgaagcttgacgacgatgtcggcctgg	pET28a-aprA-His construction
pET-PA0423-F	aagctcatatgctgaagaagacccttgc	pET42c-PA0423-His construction
pET-PA0423-R	cgctctcgagctggcgaatgccttcgacg	pET42c-PA0423-His construction
pET-lolA-F	aagctcatatgcgactgatccgcacg	pET42c-lolA-His construction
pET-lolA-R	cgctctcgagctcctggatcacgtcgacg	pET42c-lolA-His construction
16SrRNA -qPCR-F	ggcaggcctaacacatgca	Real-time PCR
16SrRNA-qPCR-R	gctgaatccaggagcaagct	Real-time PCR
rpoD -qPCR-F	aagagccgatctccatggaa	Real-time PCR
rpoD -qPCR-R	cgcccaggtgcgaatct	Real-time PCR
lasI-qPCR-F	ctgctgggcgagatgca	Real-time PCR
lasI-qPCR-R	gcctttgcgctccttgaac	Real-time PCR
rhlA-qPCR-F	gcccggacctgcaagtg	Real-time PCR
rhlA-qPCR-R	ggtcgaacagcaccacgtt	Real-time PCR
aprA-qPCR-F	aaccagaagatcaacctcaacga	Real-time PCR
aprA-qPCR-R	tcgacacattgcccttcaac	Real-time PCR
lasB-qPCR-F	cgcctgggcgagaaca	Real-time PCR
lasB-qPCR-R	gggaatcaggtaggagacgttgt	Real-time PCR

### 2.6 Protein purification

The C-terminally histidine-tagged LasB, AprA, PA0423 and LolA proteins were expressed in and purified from *Escherichia coli* Rosetta(DE3). Bacteria carrying each expression plasmid were cultured at 37°C to an OD_600_ of 0.4 in LB medium and then chilled to 16°C. Isopropyl-β-D-thiogalactopyranoside (IPTG) was added, and culture growth was continued at 16°C for 18 h. Cells were harvested by centrifugation, and the cells were resuspended in BactYeastLysis reagent (ATTO, Tokyo, Japan) and lysed by sonication. An equal volume of purification buffer (25 mM Tris [pH 7.5], 100 mM NaCl, 1 mM CaCl_2_, 2 mM dithiothreitol, and 10% glycerol) was added to the lysate, and the mixture was centrifuged. The resulting supernatant was mixed with nickel-nitrilotriacetic acid (Ni-NTA) agarose (Qiagen, Valencia, CA, USA) for 1 h. The agarose was washed twice with 10 mM imidazole and once with 50 mM imidazole, and protein was eluted with 200 mM imidazole as described previously (Hirakawa et al., 2018). The protein concentration was determined using a Bio-Rad protein assay (Bio-Rad, Hercules, CA, USA).

### 2.7 Protein adsorption assay

To test the adsorption of target proteins to MgOC_150_, each protein (10 μg) was incubated with and without MgOC_150_ for 2 h at 4 °C. Non-adsorbed protein in the supernatant was quantified in a Bio-Rad protein assay according to the Bradford method (Bio-Rad Laboratories, Hercules, CA, USA).

### 2.8 Pyocyanin assay

The pyocyanin level was determined as previously described (Essar et al., 1990; Hirakawa et al., 2021b). Bacteria were grown in LB medium for 24 h and the cell cultures were centrifuged to remove the cell pellets. Pyocyanin in the culture supernatants was extracted with chloroform and then re-extracted into 0.2 M HCl. Pyocyanin was quantified by measuring the absorbance of this solution at 520 nm.

### 2.9 Quantitative real-time PCR

The transcript levels of *lasI, rhlA, aprA* and *lasB* were measured in quantitative real-time PCR assays. Bacteria were grown with and without 30 mg MgOC_150_ in 5 mL of LB medium to the early stationary phase (OD_600_ =2.0). Total RNA was extracted using a Monarch Total RNA Miniprep Kit (New England Biolabs, Ipswich, MA, USA) following the manufacturer's instructions. cDNA synthesis and real-time PCR were carried out as previously described (Hirakawa et al., 2021a, Hirakawa et al., 2020a). The constitutively expressed 16S rRNA and *rpoD* genes were used as internal controls. The primers used for real-time PCR are listed in Table 2.

### 2.10 Promoter assay

The promoter activities of *lasI* and *rhlA* were measured as previously described (Hirakawa et al., 2021b). The PAO1 strains carrying each reporter plasmid, pBBRlasI (PAO1)-P or pBBRrhlA (PAO1)-P, were grown in the presence and absence of 30 mg MgOC_150_ in 5 mL of LB medium to the early stationary phase (OD_600_ =2.0). The chemiluminescent signal in the cell lysates was generated using a Tropix Galacto-Light Plus kit according to the manufacturer's instructions (Thermo Fisher Scientific, Waltham, MA, USA). Their β-galactosidase activities corresponding to LacZ expression were determined as the signal value normalized to an OD600 of 1.

### 2.11 Adsorption assays for antimicrobial agents

To test the adsorption of antimicrobial agents to MgOC_150_, 1.25 mg levofloxacin, aztreonam, piperacillin, cefepime and meropenem, or 5 mg amikacin, fosfomycin and colistin in 5 mL aqueous solution were incubated with and without 30 mg MgOC_150_ for 2 hours. Drug amounts were calculated as described previously (Hirakawa et al., 2020b).

### 2.12 Antimicrobial susceptibility test

To estimate the susceptibility to antimicrobial agents, MIC assay was performed according to a serial agar dilution method with the standard method of the Clinical and Laboratory Standards Institute (CLSI). The MICs were determined as the lowest concentration at which growth was inhibited. Amikacin, fosfomycin and colistin were incubated with and without MgOC_150_ for 2 hours. After removal of MgOC_150_ by centrifugation at 15,000 g, the supernatants were used for the MIC assay.

### 2.13 Statistical analysis

The *p*-value in each assay was determined using the unpaired t-test for two-group comparison between control and MgOC_150_-treated samples and the one-way ANOVA for multigroup comparison among samples treated with different amounts of MgOC_150_ / AST-120. An alpha value of 0.05 was chosen as our significance cut-off for all analyses.

## 3 Results

### 3.1 MgOC_150_ attenuates cytotoxicity of *P. aeruginosa* in intestinal epithelial cells

**Fig. 1:**
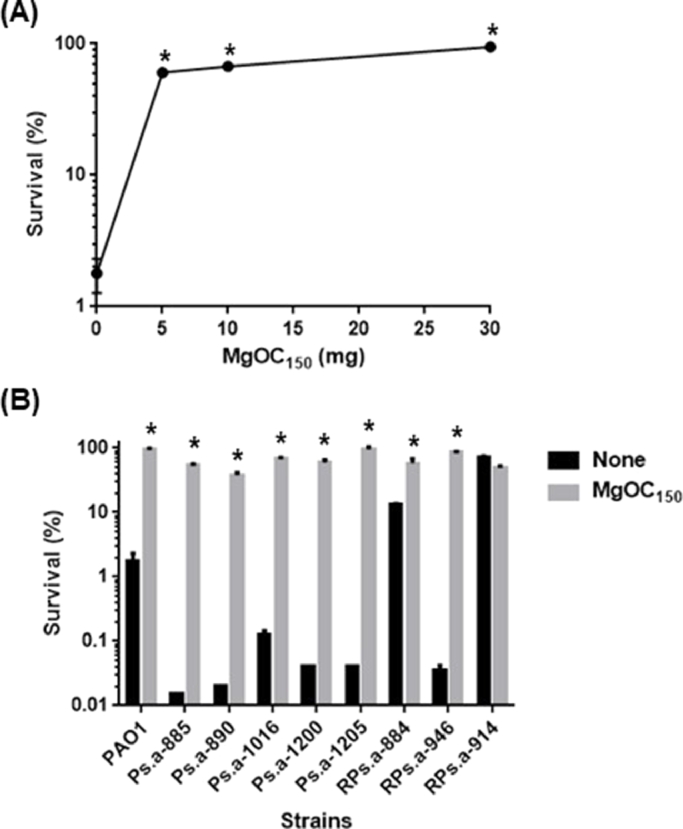
Cytotoxicity of supernatants from *P. aeruginosa* PAO1 strain and drug-resistant clinical isolates. (A) Survival of Caco-2 cells after incubation with culture supernatants from PAO1 cultured with MgOC_150_. (B) Survival of Caco-2 cells after incubation with culture supernatants from the indicated *P. aeruginosa* strains cultured in the presence or absence of 30 mg MgOC_150_. The survival rates are presented as the percentage of the RLU value for the cells after incubation with each supernatant relative to that after incubation without supernatant. Data plotted are the means of three biological replicates, and error bars indicate the standard deviations. Asterisks denote significance for values (p < 0.05) of survival rates in CaCO-2 cells after incubated with supernatant samples from bacterial cultures in the presence of MgOC_150_ relative to those after incubation with supernatant samples from bacterial cultures in the absence of MgOC_150_. The experiments were repeated twice with similar results.

**Fig. 2:**
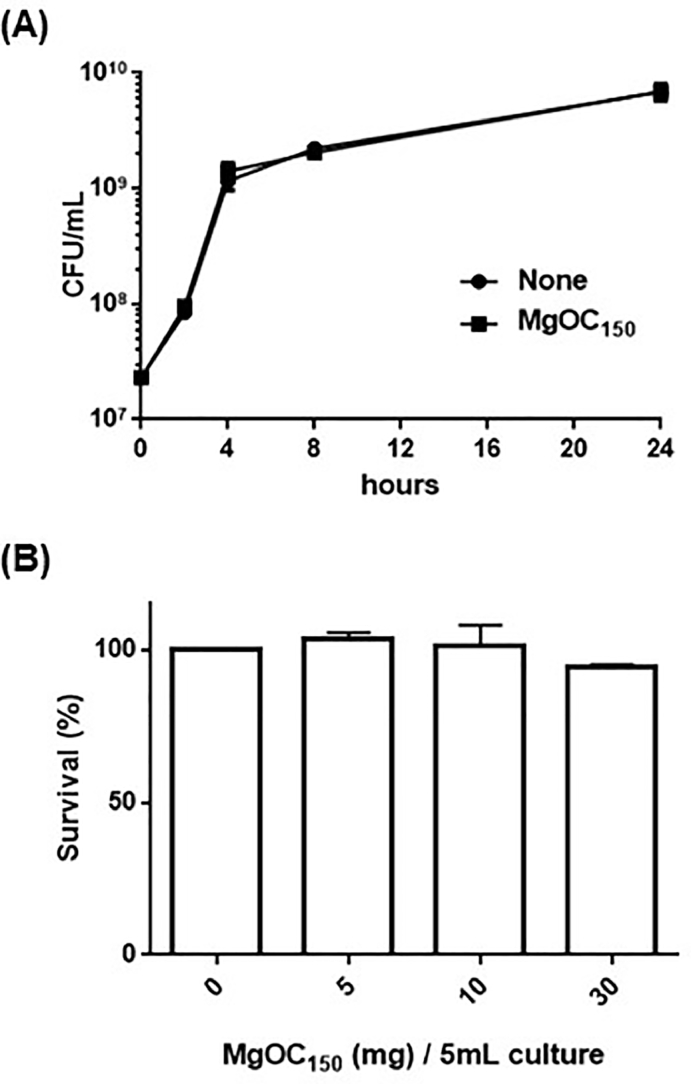
Toxicity of MgOC_150_ in bacterial and host cells. (A) Growth of the PAO1 strains cultured with and without 30 mg MgOC_150._ Bacterial growth was estimated by measuring the CFUs. (B) Survival of Caco-2 cells after incubation with and without MgOC_150_. MgOC_150_ was added into 150 μL Caco-2 cell cultures. Amounts (mg) of MgOC_150_ on the *x*-axis were represented as those corresponding to 5 mL cultures. The survival rates are presented as the percentage of the RLU value for the cells after incubation with MgOC_150_ relative to that after incubation without MgOC_150_. Data plotted are the means of three biological replicates, and error bars indicate the standard deviations. The experiments were repeated twice with similar results. No statistical significance for values of samples treated with and without MgOC_150_ was detected.

*P. aeruginosa* produces many extracellular protein molecules, and some of them target and impair host epithelial cells. First, to evaluate an efficacy of MgOC_150_ that attenuates virulence of *P. aeruginosa* against intestinal epithelial cells, the toxicity of supernatants from *P. aeruginosa* PAO1 cultured with and without MgOC_150_ to human colon adenocarcinoma Caco-2 cells was determined. The addition of supernatant from the PAO1 culture killed ∼98 % of Caco-2 cells, while more than 60 % and 90 % of the host cells survived even after incubated with supernatant from PAO1 cultured in the presence of 5 and 30 mg MgOC_150_, respectively (Fig. 1A). The capability of MgOC_150_ to neutralize cytotoxicity in culture supernatants from drug-resistant clinical isolates, including multidrug resistant *P. aeruginosa* (MDRP) strains, was also tested. Supernatants from Ps.a-885, Ps.a-890, Ps.a-1016, Ps.a-1200, Ps.a-1205, Rps.a-884 and RPs.a-946, but not from RPs.a-914, exhibited the strong cytotoxic activity against Caco-2 cells . The addition of MgOC_150_ into their cultures highly reduced the cytotoxicity (Fig. 1B). MgOC_150_ did not impair bacterial growth because there was no significant difference in bacterial CFUs when PAO1 was cultured in the presence and absence of MgOC_150_ (Fig. 2A). These results suggest that MgOC_150_ attenuates the toxicity of *P. aeruginosa* culture to intestinal epithelial cells, but it does not affect bacterial growth.

To confirm that MgOC_150_ does not disturb Caco-2 cells, the host cells were incubated with the MgOC_150_ material for 24 h. No significant reduction in the number of viable cells was observed after incubation with MgOC_150_ (Fig. 2B).

### 3.2 MgOC_150_ decreases extracellular proteolytic activity in *P. aeruginosa* cultures

**Fig. 3:**
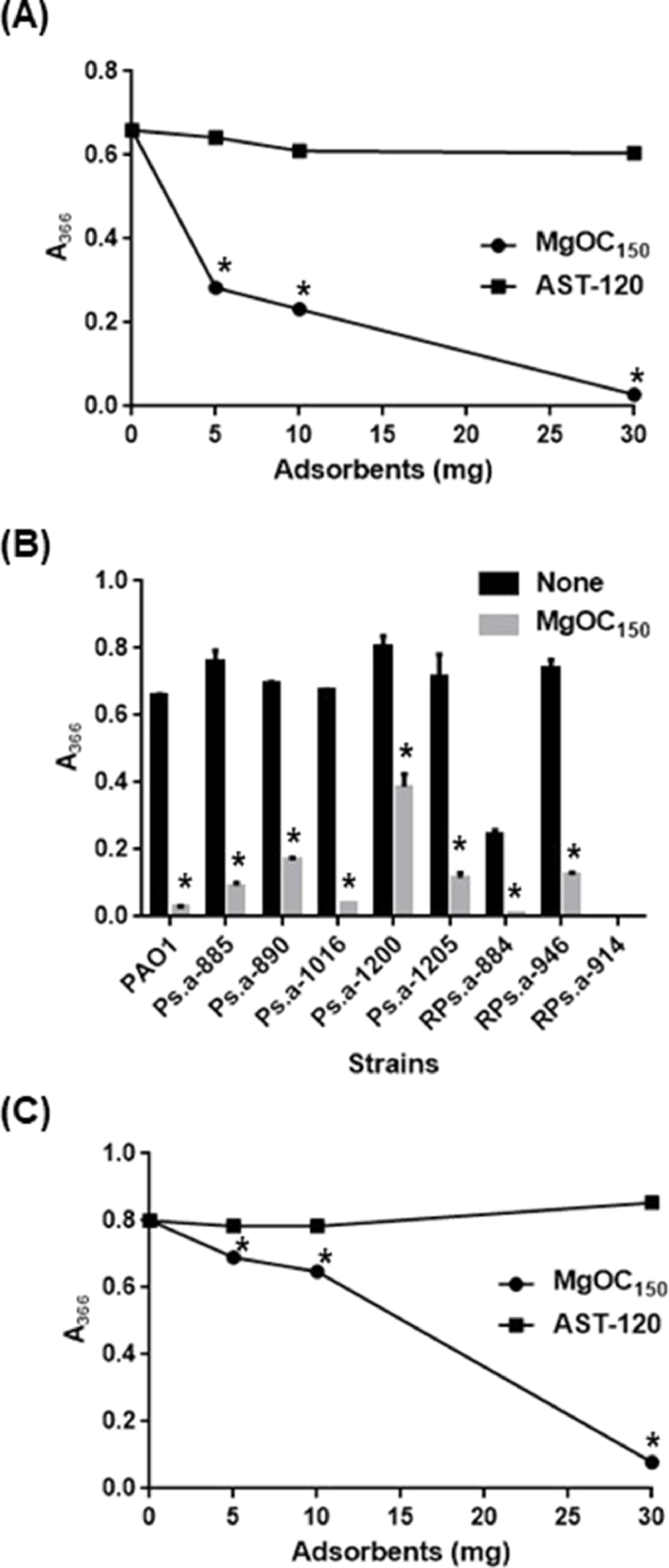
Proteolytic activities and adsorption of extracellular proteases produced by *P. aeruginosa*. (A) Extracellular protease level corresponding to the absorbance of 366 nm in the PAO1 culture grown with and without MgOC_150_ / AST-120. (B) Extracellular protease level corresponding to the absorbance of 366 nm in the PAO1 strain and *P. aeruginosa* drug-resistant clinical isolates grown with and without 30 mg MgOC_150_. (C) Extracellular protease level corresponding to the absorbance of 366 nm in supernatant from the PAO1 culture after incubation for 2 h with or without MgOC_150_ / AST-120. Data plotted are the means of three biological replicates, and error bars indicate the standard deviations. Asterisks denote significance for values (p < 0.05) of samples or bacterial cultures in the presence of MgOC_150_ relative to those in the absence of MgOC_150_. The experiments were repeated twice with similar results.

A subset of extracellular proteases, produced by *P. aeruginosa* are involved in pathogenesis of this bacterium. Attenuated cytotoxicity of a culture supernatant from *P. aeruginosa* cultured with MgOC_150_ may be due to a reduced extracellular proteolytic activity. To test this hypothesis, the proteolytic activity in a supernatant from the bacterial culture was assessed by measuring their abilities to degrade azocasein as a proteolytic substrate. A degradation product of azocasein was detected at an absorbance of 366 nm when incubated with a supernatant from the PAO1 culture. On the other hand, when azocasein was incubated with a supernatant from bacteria co-cultured with 30 mg MgOC_150,_ its degradation product was undetectable (Fig. 3A). To compare the capability of MgOC_150_ with that of AST-120 used as an oral adsorbent medicine, the PAO1 strain was cultured with 30 mg AST-120. In contrast to MgOC_150_, a significant level of the proteolytic activity was still observed in the supernatant from the bacteria cultured with AST-120 (Fig. 3A). In addition to PAO1, supernatants from Ps.a-885, Ps.a-890, Ps.a-1016, Ps.a-1200, Ps.a-1205, RPs.a-884, and RPs.a-946 cultures exhibited proteolytic activities, and their activities was reduced by adding MgOC_150_ into cultures although a moderate proteolytic activity remained (Fig. 3B). Unlike those from PAO1, Ps.a-885, Ps.a-890, Ps.a-1016, Ps.a-1200, Ps.a-1205, RPs.a-884, and RPs.a-946, the proteolytic activity of the supernatant from RPs.a-914 was below the limit of detection even when cultured in the absence of MgOC_150_. Therefore, low cytotoxicity of the RPs.a-914 culture may be due to its deficient proteolytic activity. To estimate the ability of MgOC_150_ to adsorb proteolytic activity-associated proteins, the bacteria-free supernatant from a 24 h culture of PAO1 was incubated with MgOC_150_, and the residual proteolytic activity in the supernatant after removal of MgOC_150_ was determined. The proteolytic activity decreased by ∼90% after incubation with 30 mg MgOC_150_ (Fig. 3C). The bacteria-free supernatant was also incubated with AST-120. The proteolytic activity in the supernatant did not decrease (Fig. 3C). Altogether, these results suggest that MgOC_150_ adsorbs some extracellular proteins of *P. aeruginosa*, including extracellular proteases, and decreases their proteolytic activity in bacterial culture.

### 3.3 Addition of MgOC_150_ decreases levels of certain extracellular proteins, including LasB elastase and AprA alkaline protease, in *P. aeruginosa* culture

**Fig. 4:**
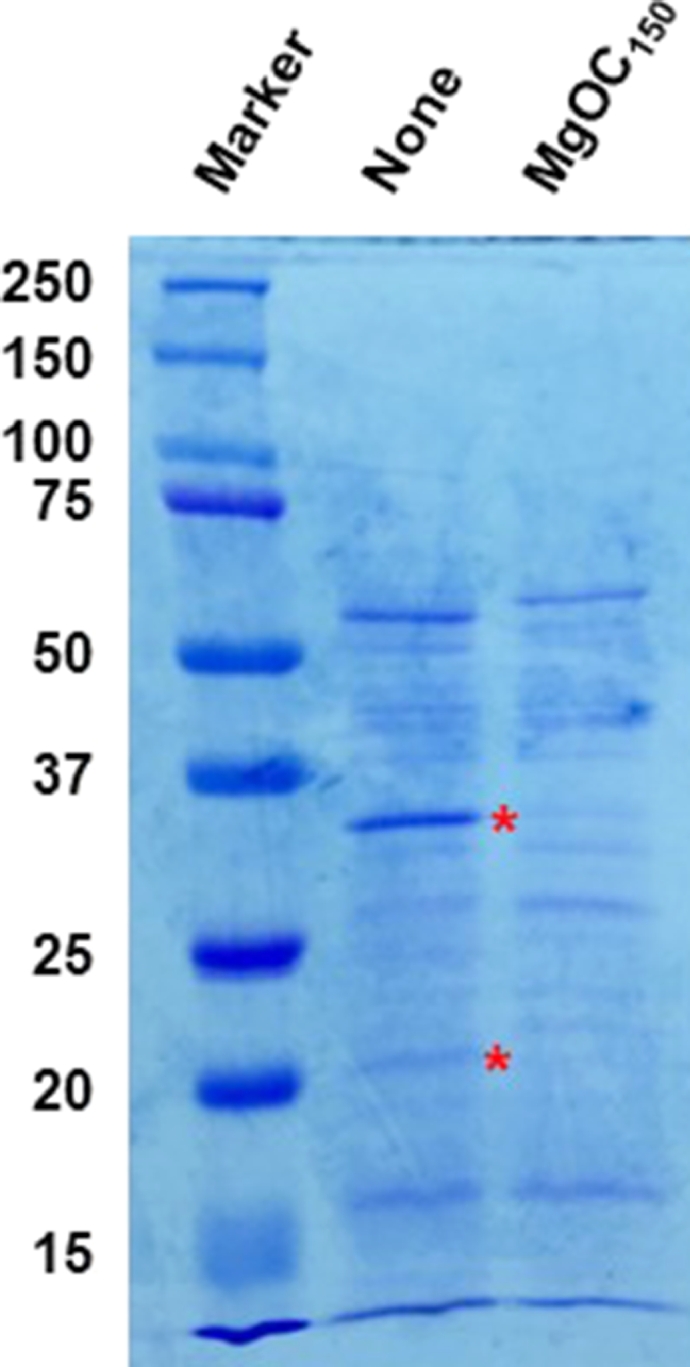
Extracellular proteins from the PAO1 strain cultured in the presence and absence of MgOC_150_. Proteins were separated by SDS-PAGE using a 10% acrylamide gel, and stained with Coomassie brilliant blue. Locations of molecular mass standards (in kDa) are indicated on the left. Asterisks indicate protein bands which levels are remarkably low in the strain cultured in the presence of MgOC_150_ compared to the strain cultured in the absence of MgOC_150_.

To identify extracellular proteins of *P. aeruginosa*, that are adsorbed by MgOC_150_, PAO1 strains were cultured in the presence and absence of MgOC_150_, and proteins in culture supernatants were analyzed using a proteomics technique. Proteins were separated by SDS-PAGE and stained with CBB. Two major protein bands, corresponding to ∼35 and 20 kDa, respectively, had intensities that were remarkably lower in the supernatant from PAO1 grown with MgOC_150_ compared to the supernatant from PAO1 grown without MgOC_150_ (Fig. 4). Proteins contained in these bands were identified (The dataset ID is PXD032870 in the ProteomeXchange Consortium). Table 3 and Table 4 show the top 35 highest-scoring candidate proteins, which were identified from either 35 or 20 kDa bands detected by SDS-PAGE from the culture supernatant of PAO1. Firstly, we note that some proteins were identified in both fractions that differ in molecular weight. We guess that these proteins were detected as partial degradation products. Two extracellular proteases involved in the pathogenesis of *P. aeruginosa*, predicted LasB elastase and AprA alkaline protease (Cryz and Iglewski, 1980; Kessler and Safrin, 2014), were detected for the 35 kDa band (Table 3). LasB is produced as a 54 kDa precursor protein and it is processed to a 33 kDa active form when secreted into the extracellular space (Kessler and Safrin, 1988a, Kessler and Safrin, 1988b). Therefore, it is reasonable that the peptides corresponding to this protein were detected in the 35 kDa fraction. In contrast, the predicted molecular size of AprA is 50 kDa and detected AprA peptides may be derived from its partial degradation product. Comparative quantitative analysis of these proteins in samples with or without MgOC_150_ incubation were also performed using MaxQuant software (indicated by LFQ intensity in Tables 3 and 4). Although LasB was detected in both samples, the LFQ intensity of LasB detected in the 35 kDa band from the supernatant of PAO1 grown with MgOC_150_ was approximately 13-fold lower than that grown without MgOC_150_ (Table 3). In contrast, AprA was only identified from the supernatant of PAO1 grown without MgOC_150_, and the LFQ intensity of AprA from PAO1 grown with MgOC_150_ was nearly zero (Table 3). These results suggest that levels of extracellular LasB and AprA decreased when cultured with MgOC_150_.

**Table 3:** Top 35 protein candidates in scores for the 35 kDa fraction from the culture supernatant of PAO1

Uniprot ID	Proteins	Score	LFQ intensity	LFQ intensity
			None*1	MgOC_150_⁎2
P30718	Chaperonin (GroEL)	259.55	1.03.E+08	1.22.E+08
P72151	B-type flagellin (FliC)	238.66	1.60.E+08	8.01.E+07
Q9I4P3	Flagellar hook-associated protein (FlgK)	222.17	2.06.E+08	0.00.E+00
P21175	Branched-chain amino acid transport protein (BraC)	176.33	3.10.E+07	1.45.E+08
Q9I402	L-glutamate/L-aspartate-binding protein (PA1342)	161.24	3.78.E+07	6.34.E+07
Q9HXG8	Uncharacterized protein (PA3836)	156.33	8.59.E+07	1.34.E+08
Q9I3D1	Dihydrolipoyl dehydrogenase (IpdG)	155.15	8.33.E+06	2.51.E+07
Q9I047	Transaldolase (Tal)	154.3	4.43.E+07	7.20.E+07
Q9HV43	Chaperone protein (DnaK)	151.49	1.99.E+07	1.07.E+07
P14756	Elastase (LasB)	150.19	2.23.E+09	1.75.E+08
Q9K3C5	B-type flagellar hook-associated protein (FliD)	143.91	2.38.E+07	6.99.E+04
Q9HU18	C4-dicarboxylate-binding protein (DctP)	126.13	3.13.E+07	4.94.E+07
Q9HVA2	Ketol-acid reductoisomerase (IlvC)	124.36	3.01.E+07	9.08.E+07
Q9I0D3	Cysteine synthase (CysK)	124.14	3.51.E+07	9.66.E+07
Q9HZQ8	Aminopeptidase (Lap)	122.34	9.27.E+07	0.00.E+00
Q9I3C5	Chaperone protein (HtpG)	121.69	1.64.E+07	0.00.E+00
G3XDA8	Phosphate-binding protein (PstS)	118.77	2.37.E+07	3.28.E+07
Q59653	Aspartate carbamoyltransferase (PyrB)	116.56	9.58.E+06	1.67.E+07
P13794	Outer membrane porin F (OprF)	109.31	2.11.E+07	4.88.E+07
Q9HTX3	Iron transporter (PA5217)	107.35	8.89.E+07	1.90.E+08
G3XCX5	Bacteriophage protein (PA0618)	105.36	2.09.E+08	1.50.E+08
Q9I5D1	AmpDh3	104.79	1.40.E+06	1.64.E+07
Q9HZ48	ABC sugar transport protein (PA3190)	102.02	8.33.E+05	9.45.E+06
Q9I5W4	Immunomodulating metalloprotease (ImpA)	101.99	1.04.E+07	1.01.E+07
Q9HZ71	30S ribosomal protein S1 (RpsA)	100.1	1.10.E+07	0.00.E+00
Q9HVN5	Chaperone protein (ClpB)	99.932	2.89.E+07	0.00.E+00
Q9HT29	Uncharacterized protein (PA5545)	99.795	8.54.E+06	2.67.E+07
Q9I7A7	LysM domain-containing protein (PA0020)	96.521	3.13.E+05	1.05.E+07
Q9I5Q3	Tyrosine–tRNA ligase 2 (TyrS2)	95.356	1.48.E+07	0.00.E+00
O86428	Branched-chain-amino-acid aminotransferase (IlvE)	92.5	6.74.E+07	9.91.E+07
Q03023	Alkaline protease (AprA)	90.26	2.30.E+07	0.00.E+00
Q9I456	Outer membrane protein (PA1288)	81.569	4.94.E+06	1.18.E+07
Q9I650	Virginiamycin B lyase (PA0468)	79.946	1.24.E+07	2.08.E+07
Q9I589	Chitin-binding protein (CbpD)	77.539	1.38.E+08	1.70.E+07
O82851	Elongation factor Ts (Tsf)	76.463	1.47.E+07	1.14.E+07

*1 : Proteins from a supernatant of PAO1 grown without MgOC_150_

⁎2 : Proteins from a supernatant of PAO1 grown with MgOC_150_

**Table 4:** Top 35 protein candidates in scores for the 20 kDa fraction from the culture supernatant of PAO1

Uniprot ID	Proteins	Score	LFQ intensity	LFQ intensity
			None*1	MgOC_150_⁎2
Q9HV43	Chaperone protein (DnaK)	239.49	1.73.E+08	0.00.E+00
Q9K3C5	B-type flagellar hook-associated protein (FliD)	204.01	2.53.E+08	3.18.E+06
P72151	B-type flagellin (FliC)	183.15	6.00.E+07	2.54.E+07
P30718	Chaperonin (GroEL)	181.64	2.71.E+07	3.54.E+07
Q9I690	Extracellular protease ( PA0423)	162.39	7.75.E+08	1.89.E+09
P14756	Elastase (LasB)	147.53	2.09.E+08	4.93.E+07
Q9I0M4	Outer membrane lipoprotein carrier protein (LolA)	141.3	1.76.E+08	1.77.E+08
O82853	Ribosome-recycling factor (Frr)	131.5	7.45.E+07	1.02.E+07
Q9I5D1	AmpDh3	128.06	2.78.E+07	1.02.E+08
P13794	Outer membrane porin F (OprF)	126.26	8.62.E+07	1.23.E+08
Q9I2F8	D-Ribose/D-allose-binding protein (RbsB)	120.15	6.37.E+06	4.19.E+07
G3XD47	Arginine/ornithine binding protein (AotJ)	107.83	2.10.E+07	2.55.E+07
Q9I4Y4	Pyocin S5 (PyoS5)	106.84	3.93.E+07	0.00.E+00
Q9HTM5	Uncharacterized protein (PA5330)	101.42	4.72.E+07	3.21.E+07
Q9HZQ8	Aminopeptidase (Lap)	99.454	7.51.E+07	0.00.E+00
Q9I3D1	Dihydrolipoyl dehydrogenase (IpdG)	98.982	1.36.E+07	2.53.E+07
Q9I402	L-Glutamate/L-aspartate-binding protein (PA1342)	98.45	1.49.E+07	2.45.E+07
P21175	Branched-chain amino acid transport protein (BraC)	98.406	2.36.E+06	3.18.E+07
Q9HVA8	Ferric iron-binding protein (HitA)	96.294	2.87.E+06	1.21.E+07
P08308	Ornithine carbamoyltransferase (ArcB)	94.575	1.95.E+06	1.35.E+07
Q9HXL0	Uncharacterized protein (PA3785)	91.92	3.26.E+08	2.08.E+08
Q9I3D8	Uncharacterized protein (PA1579)	89.453	1.62.E+08	7.23.E+07
Q9I456	Outer membrane protein (PA1288)	76.379	5.59.E+06	8.98.E+06
G3XD39	Bacteriophage protein (PA0622)	75.407	2.48.E+07	1.72.E+07
Q9HVV7	LPS export protein (LptA)	75.126	2.09.E+07	1.50.E+07
Q9HY81	Alkyl hydroperoxide reductase C (PA3529)	72.492	2.80.E+08	6.63.E+05
Q9I4P3	Flagellar hook-associated protein (FlgK)	68.059	1.33.E+07	0.00.E+00
G3XD52	MaoC-like protein (PA3302)	66.561	3.27.E+07	3.04.E+07
Q9HU31	ABC transporter periplasmic binding protein (PA5153)	63.569	1.26.E+06	1.45.E+07
Q9I1R3	ABC transport protein (PA2204)	62.183	1.02.E+06	5.00.E+06
Q9I6J1	Putrescine-binding protein (SpuD)	61.373	3.67.E+06	5.48.E+06
Q9I5W4	Metalloprotease (ImpA)	61.116	1.17.E+07	9.64.E+06
Q9HW11	UPF0234 protein (PA4395)	59.911	3.33.E+07	1.58.E+06
Q9I457	Glutathione peroxidase (PA1287)	59.694	1.08.E+08	1.14.E+08
Q9HV46	Transcription elongation factor (GreA)	58.009	1.26.E+07	4.30.E+06

*1 : Proteins from a supernatant of PAO1 grown without MgOC150

⁎2 : Proteins from a supernatant of PAO1 grown with MgOC150

LasB was also detected from the 20 kDa band, which may be processed by partial degradation, and showed 4.2-fold decrease in PAO1 grown with MgOC_150_ (LFQ intensity: 2.09×10^8^ for bacteria grown without MgOC_150_ and 4.93×10^7^ for that grown with MgOC_150_, Table 4). In addition, the LasA level also decreased when cultured with MgOC_150_. The LFQ intensity of LasA detected in the 20 kDa band was more than 200-fold lower in PAO1 grown with MgOC_150_ than that grown without it (LFQ intensity: 7.23×10^7^ for bacteria grown without MgOC_150_ and 3.27×10^5^ for that grown with MgOC_150_), although LasA was identified by very low score (score 22) and not listed in the top 35 protein candidates (See PXD032870 in the ProteomeXchange Consortium). LasA is a known elastase involved in the pathogenesis of *P. aeruginosa*, and it is processed to a 22 kDa active form by LasB (Li and Lee, 2019). Thus, the decreased level of active LasA in bacteria grown with MgOC_150_ may be associated with a reduction of LasB.

Several intracellular and outer membrane-associated proteins, including GroEL, DnaK, FliC and FliD, were also detected from the 35 and 20 kDa bands (Table 3 and Table 4). Since these proteins are abundantly expressed in bacteria, significant amounts of proteins derived from lysed cells might be undesirably detected even if the lysed cell populations were low (Roncarati and Scarlato, 2017, Zhang et al., 2007).

Extracellular flagella-associated proteins of *P. aeruginosa* damage epithelial cells by inducing inflammatory responses (Haiko and Westerlund-Wikstrom, 2013). We found that FlgK protein was detected from both 35 and 20 kDa fractions in PAO1 grown without MgOC_150_ while the LFQ intensity of FlgK exhibited zero in PAO1 grown with MgOC_150_ (Table 3 and Table 4). These observations indicate that MgOC_150_ can also reduce the level of extracellular FlgK.

### 3.4 LasB and AprA proteolytic activities disrupt intestinal epithelial cells and MgOC_150_ adsorbs these proteins

**Fig. 5:**
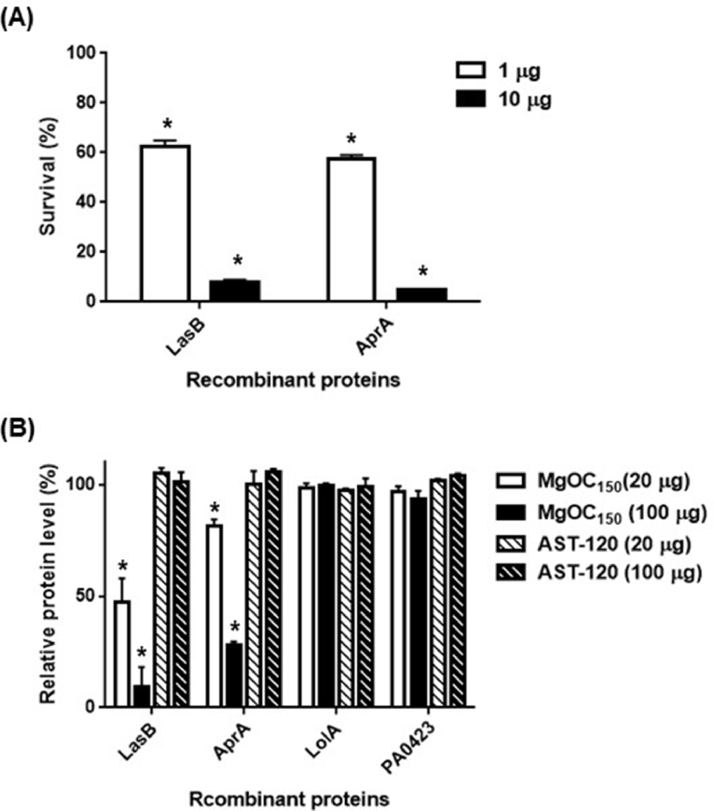
Cytotoxicity and adsorption of the recombinant LasB and AprA proteins. (A) Survival of Caco-2 cells after incubation with the purified recombinant LasB and AprA proteins. (B) The recombinant proteins (10 μg) were incubated with and without MgOC_150_ / AST-120. Relative (Non-adsorbed) protein levels are presented as the percentage of the value for samples after incubation with MgOC_150_ / AST-120 relative to that after incubation without MgOC_150_ / AST-120. Data plotted are the means from three independent experiments; and error bars indicate the standard deviations. Asterisks denote significance for values (*p* < 0.05) of the protein level after incubation with MgOC_150_ relative to that after incubation without MgOC_150_.

Reduced levels of extracellular LasB and AprA in *P. aeruginosa* grown with MgOC_150_ may be due to adsorption of these proteins. To test this hypothesis, the ability of MgOC_150_ to adsorb LasB and AprA was estimated using the C-terminally histidine-tagged recombinant proteins. First, this study demonstrated that the purified recombinant LasB and AprA proteins have protease activity and cytotoxicity in Caco-2 cells (Fig.5A and data not shown). A proteolytic product from azocasein was observed after incubation with the LasB and AprA proteins (data not shown) and administration of these proteins significantly reduced the viability of Caco-2 cells (Fig. 5A). The LasB and AprA proteins were incubated with different amounts of MgOC_150_ for 2 h, and the residual amount in the supernatant was measured after MgOC_150_ removal. More than 85 % of the LasB protein (10 μg) in a solution could be removed with MgOC_150_ when incubated with 100 μg of this adsorbent while approximately 70 % of the AprA protein was removed by adsorption to MgOC_150_ (Fig. 5B). These results suggest that MgOC_150_ adsorbs the LasB and AprA proteins and reduces the proteins-associated proteolytic activity and cytotoxicity. For the negative control, LolA and PA0423 were used from the 20 kDa fraction because their LFQ intensity was not reduced even when MgOC_150_ was added in the culture (Table 4). The C-terminally histidine-tagged LolA and PA0423 proteins were purified and adsorption of these proteins to MgOC_150_ was evaluated. Consistent with the data from the LFQ intensity, adsorption of neither the recombinant LolA nor PA0423 protein to MgOC_150_ was observed (Fig. 5B). To compare the ability of AST-120 to adsorb LasB and AprA proteins with that of MgOC_150_, these recombinant proteins were incubated with AST-120. In contrast to MgOC_150_, no adsorption of LasB and AprA to AST-120 was observed (Fig. 5B).

### 3.5 MgOC_150_ adsorbs pyocyanin and reduces its level in *P. aeruginosa* culture

**Fig. 6:**
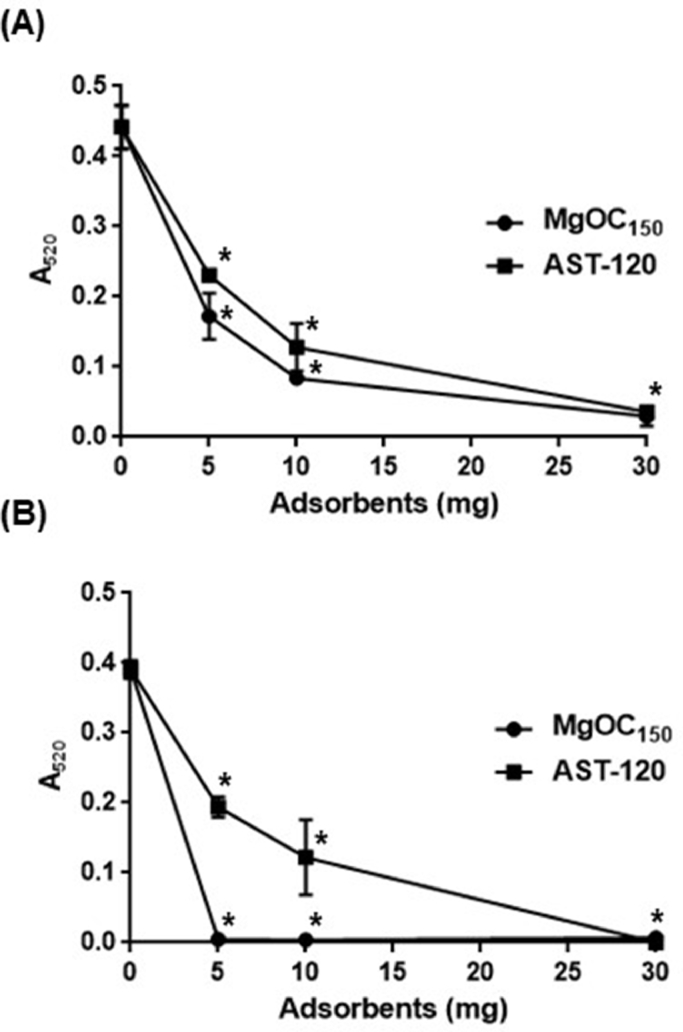
Adsorption and accumulation of pyocyanin. (A) The pyocyanin level corresponding to the absorbance of 520 nm in a supernatant from the PAO1 culture after incubation for 2 h with and without MgOC_150_ / AST-120. (B) The pyocyanin level corresponding to the absorbance of 520 nm in the PAO1 culture grown with and without MgOC_150_ / AST-120. Data plotted are the means of three biological replicates, and error bars indicate the standard deviations. Asterisks denote significance for values (*p* < 0.05) of samples or bacterial cultures in the presence of MgOC_150_ / AST-120 relative to those in the absence of MgOC_150_ / AST-120. The experiments were repeated twice with similar results.

Pyocyanin is another molecule that contributes to the virulence of *P. aeruginosa*. Its cytotoxic function has been defined in airway epithelial cells, such as A-549, but it is known to disrupt intestinal epithelial cells (Hall et al., 2016, Hirakawa et al., 2021b). Unlike extracellular proteases, pyocyanin is a non-protein small-sized molecule. We previously found that AST-120 highly adsorbs the pyocyanin molecule (Hirakawa et al., 2021b). To compare the adsorption ability of MgOC_150_ to pyocyanin with that of AST-120, the bacteria-free supernatant from a 24 h culture of PAO1 was incubated with MgOC_150_ and AST-120 for 2 h. After removal of these adsorbents, residual pyocyanin in the supernatant was quantified. Similar to AST-120, MgOC_150_ adsorbed pyocyanin in the PAO1 culture supernatant. More than 50 % and 90 % of pyocyanin could be eliminated when incubated with 5 mg and 30 mg MgOC_150_, respectively (Fig. 6A). The PAO1 strain was cultured in the presence and absence of AST-120 or MgOC_150_, and the pyocyanin level in the supernatants was measured. The pyocyanin level was below the limit of detection when cultured with the 30 mg of adsorbents (Fig. 6B). Pyocyanin was undetectable even when 5 mg MgOC_150_ was present although a significant level of pyocyanin was still observed when grown with 5 mg AST-120. Thus, MgOC_150_ can adsorb pyocyanin more effectively than AST-120.

### 3.6 MgOC_150_ does not affect the activity of LasI-LasR and RhlI-RhlR- mediated quorum sensing

**Fig. 7:**
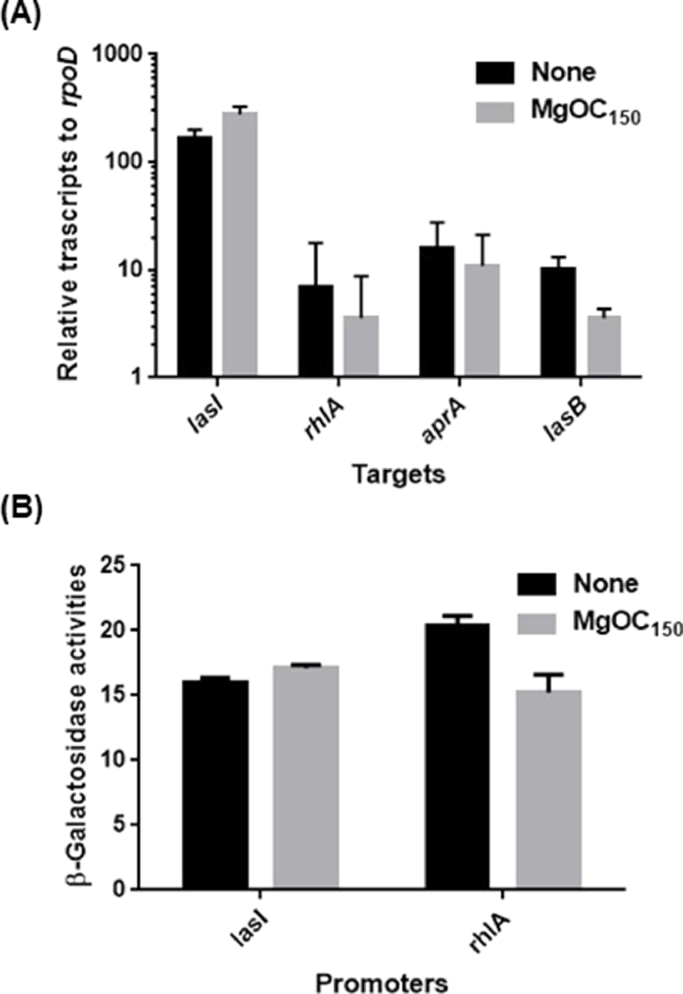
Expression of the LasI-LasR and RhlI-RhlR QS system-related genes. (A) Transcript levels of *lasI, rhlA, aprA* and *lasB* were described as values relative to that of *rpoD* (housekeeping gene). (B) Promoter levels of *lasI* and *rhlA* genes were represented by the β-galactosidase activities of PAO1 carrying pBBRlasI (PAO1)-P or pBBRrhlA (PAO1)-P, the *lacZ* reporter plasmid. Data plotted are the means of three biological replicates, and error bars indicate the standard deviations. No statistical significance for values of samples treated with and without MgOC_150_ was detected.

*P. aeruginosa* controls subsets of genes via quorum sensing (QS) that is one manner of bacterial signal transduction. This bacterium possesses two major systems mediated by LasI-LasR and RhlI-RhlR (Schuster et al., 2003). The production of LasB, AprA and pyocyanin is induced by these QS systems (Smith and Iglewski, 2003). LasI-LasR and RhlI-RhlR primarily activate the transcription of *lasI* and *rhlA* genes, respectively. The levels of *lasI* and *rhlA* transcripts were measured by qPCR analyses to estimate LasI-LasR and RhlI-RhlR activities in PAO1 grown with and without MgOC_150_. No significant difference in these transcript levels in the PAO1 strains grown with and without MgOC_150_ was seen (Fig. 7A). The levels of *lasI* and *rhlA* promoters were also measured. Consistent with the qPCR results, there was no significant difference in β-galactosidase levels corresponding to the promoter activities of *lasI* and *rhlA* between bacteria grown with and without MgOC_150_ (Fig. 7B). Thus, MgCO_150_ does not affect the activities of LasI-LasR and RhlI-RhlR-mediated QS systems associated with the production of LasB, AprA and pyocyanin. There was also no significant difference in the *aprA* transcript level in PAO1 grown with and without MgOC_150_. However, the *lasB* level in PAO1 grown with MgOC_150_ was moderately lower than that in PAO1 grown without MgOC_150_ (Fig. 7A).

### 3.7 MgOC_150_ does not impair antibacterial activity of amikacin, fosfomycin and colistin

**Fig. 8:**
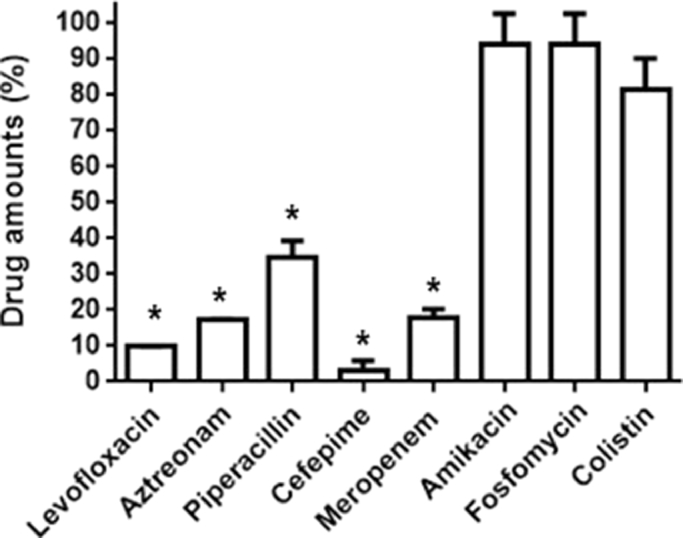
Drug adsorption by MgOC_150_.Each drug was incubated in an aqueous solution with and without 30 mg MgOC_150_ for 2 hours. The *y* axis shows the percent of drug amount (mg) after incubation with MgOC_150_ relative to the drug amount (mg) after incubation without MgOC_150_. Data plotted are the means from three independent experiments; error bars indicate the standard deviations. Asterisks denote significance for values (*p* < 0.05) of drug amount after incubation with MgOC_150_ relative to that after incubation without MgOC_150_.

To investigate whether MgOC_150_ can be used together with antimicrobial agents, we estimated the capability of MgOC_150_ to adsorb some classes of drugs that are commonly used for treatment of *P. aeruginosa* infections. Levofloxacin, aztreonam, piperacillin, cefepime and meropenem were highly adsorbed by MgOC_150_ because these drugs were eliminated more than 60% after incubation with MgOC_150_ (Fig. 8). In contrast, amikacin, fosfomycin and colistin retained more than 75% even after incubation with MgOC_150_ (Fig. 8). We determined that MICs of amikacin fosfomycin and colistin were 2, 64 and 32 mg/L in PAO1 and >64, 64 and 16 mg/L in RPs.a-884, respectively (Table 5). After incubation with MgOC_150_, MICs of amikacin, fosfomycin, and colistin exhibited 2, 64 and 32 mg/L in PAO1 and >64, 64 and 32 mg/L in RPs.a-884, respectively (Table 5). These results suggest that MgOC_150_ does not impair the activities of amikacin fosfomycin and colistin.

**Table 5:** Amikacin, fosfomycin, and colistin MICs of *P. aeruginosa* PAO1 and RPs.a-884 strains

Drugs	MICs (mg/L)
	PAO1 RPs.a-884
Amikacin	2 >64
Amikacin + MgOC_150_*	2 >64
Fosfomycin	64 64
Fosfomycin + MgOC_150_*	64 64
Colistin	32 16
Colistin + MgOC_150_*	32 32

⁎ Drugs were pre-treated with 30 mg of MgOC150 in 5 mL aqueous solutions for 2h, and used for MIC assays.

## 4 Discussion

*Pseudomonas*-caused bacteremia is commonly hospital-acquired and is a problem worldwide. It can result from some sources including primary infection in the lungs, gastrointestinal tract, urinary tract, skin, and soft tissue (Turner et al., 2014, Mittal et al., 2009, Zaborina et al., 2006). It is extremely hard to prevent bacteremia caused by intestinal *P. aeruginosa* because they cannot be selectively eliminated without killing other normal gut bacteria.

Several adsorbents have been approved as oral medicines, such as colestyramine and AST-120, which are used for the treatment of the hypercholesterolemia and the pruritus in individuals with liver failure and treatment of progressive chronic kidney disease, respectively (Scaldaferri et al., 2013, Hegade et al., 2015, Niwa, 2011). However, no adsorbent medicine is approved for treatment of infectious diseases. MgOC_150_ is used for industry purposes, including an electrode catalyst, a bioelectrode, and enzyme immobilization (Funabashi et al., 2017, Tsujimura et al., 2014, Mazurenko et al., 2018). This study proposed a potential benefit of MgOC_150_ that attenuates the toxicity of intestinal *P. aeruginosa* (Fig. 1). Some extracellular molecules produced by *P. aeruginosa*, including LasB elastase, AprA alkaline protease and pyocyanin, disrupt intestinal epithelial cells serving as an intestinal barrier to prevent bacterial entry into the bloodstream (Fig. 5A, Hirakawa et al., 2021b). MgOC_150_ confers protection to the intestinal barrier function against intestinal *P. aeruginosa,* including strains that are resistant to conventional antimicrobial agents, by adsorbing these molecules (Figs. 1 and Fig. 3, Fig. 4, Fig. 5, Fig. 6, Tables 3 and 4).

MgOC_150_ is predicted to highly adsorb molecules that are more than 30 kDa (Funabashi et al., 2017). Therefore, the effective adsorption of LasB and AprA by MgOC_150_ is reasonable. In contrast to MgOC_150_, AST-120 poorly adsorbed the LasB and AprA proteins (Figs. 3 and 5B). The average pore size of AST-120 is approximately 2 nm, and this pore size may be too small to adsorb the LasB and AprA proteins, but it can highly adsorb pyocyanin (Fig. 6, Hirakawa et al., 2021b). Similar to AST-120, MgOC_150_ adsorbed pyocyanin, although the size of the pyocyanin molecule is much smaller than the pore size of MgOC_150_ (Fig. 6). The pore of MgOC_150_ is produced from an Mg-containing template substrate. If some hydroxyl groups derived from this substrate molecule remain even after the pyrolysis process, a significant amount of a small-sized compounds may be bound by this hydroxyl group. For these reasons, MgOC_150_ is a superior adsorbent compared to AST-120 for the suppression of *P. aeruginosa* virulence.

On the other hand, the adsorption ability of MgOC_150_ for LolA and PA0423 was relatively low (Table 4 and Fig. 5B). Molecular sizes of the LolA and PA0423 monomers are 23 kDa and 21 kDa, respectively, and they are smaller than the pore size of MgOC_150_.The LolA protein was shown to form a complex with lipoproteins in the periplasmic space (Takeda et al., 2003) while one zymography data showed that the PA0423 protein is secreted into the extracellular space where it forms a tetramer (Tang et al., 2009). These protein complexes may fit pores from MgOC_150_. However, the results of the proteomics analysis and adsorption experiment imply that significant amounts of the LolA and PA0423 monomers may exist in the extracellular fraction under this study's culture conditions, which may explain the low adsorption ability of MgOC_150_ for extracellular LolA and PA0423.

LasB is the most abundant extracellular protease produced by *P. aeruginosa* and its role on the pathogenesis has been characterized (Beaufort et al., 2013). This protease degrades mucins, cadherin, and surfactant proteins, which induce tissue injury and bacterial dissemination (Alcorn and Wright, 2004, Golovkine et al., 2014, Kuang et al., 2011, Mun et al., 2009). The cytotoxicity of the LasB protein is well defined in respiratory epithelial cells. This study demonstrated that the recombinant LasB protein impairs intestinal epithelial cells (Fig. 5A). The LasB protein also degrades IgG, IgA, several cytokines (including IFN-γ and IL-6), and protein molecules involved in the complement system (Bainbridge and Fick, 1989, Bastaert et al., 2018, Heck et al., 1990, Parmely et al., 1990, Saint-Criq et al., 2018). AprA is another extracellular protease contributing to the virulence of *P. aeruginosa*. Similar to LasB, AprA has the ability to modulate immune responses because it degrades TNF-α and IFN-γ together with the C3 and C2 protein complexes composing the complement system (Hong and Ghebrehiwet, 1992, Laarman et al., 2012, Parmely et al., 1990). Thus, adsorption of the LasB and AprA proteins using MgOC_150_ decreases its cytotoxicity in the gut and the ability of bacteria to evade the host immune system. AprA activity has been also shown to sustain the production of pyocyanin (Iiyama et al., 2017). Therefore, adsorption of the AprA protein may lead to a decreased pyocyanin production.

Pyocyanin is a major virulence molecule associated with the progression of chronic respiratory infections, such as in patients with CF (Wilson et al., 1988). We suggest that this molecule also has an important role in the pathogenesis of intestinal *P. aeruginosa* for the following reasons. Pyocyanin damages intestinal epithelial cells because the *phzA1/B1* mutant that does not produce pyocyanin is much less toxic in Caco-2 cells than the pyocyanin-producing parent PAO1 strain (Hirakawa et al., 2021b). This molecule also contributes to anaerobic survival, iron acquisition, and biofilm development (Costa et al., 2015, Glasser et al., 2014, Wang et al., 2011). Available oxygen and iron for intestinal *P. aeruginosa* could be limited in the gut by microorganism competitors while biofilm formation enables *P. aeruginosa* to evade many antimicrobial compounds and intestinal immune systems (Ciofu and Tolker-Nielsen, 2019). Therefore, pyocyanin may support the colonization and survival of *P aeruginosa* in the gut. MgOC_150_ can eliminate the benefits conferred by pyocyanin for intestinal *P. aeruginosa*. Additionally, pyocyanin has a bactericidal activity against many gut microbiota which act as an intestinal barrier. One study suggested that *E. coli* protects mice from intestinal *P. aeruginosa* (Christofi et al., 2019). Thus, adsorption of pyocyanin by MgOC_150_ may protect the host from intestinal *P. aeruginosa* by alleviating damage to intestinal epithelial cells and disturbance of gut microbiota, including *E. coli,* contributing to the homeostasis of the intestinal barrier.

The production of LasB, AprA and pyocyanin is induced by LasI-LasR and RhlI-RhlR-mediated QS systems because strains that lack these QS activities do not produce these molecules adequately (Gambello et al., 1993, Pearson et al., 1997, Van Delden and Iglewski, 1998). LasB, AprA and pyocyanin-associated virulence can be attenuated by disrupting these QS systems. For this reason, interference of QS is noticed as a promising alternative antimicrobial strategy (Hirakawa and Tomita, 2013, Whiteley et al., 2017). Since MgOC_150_ does not affect the QS systems (Fig. 7), it may be incorporated with novel approaches to prevent and treat *P. aeruginosa* infections.

Undesirably, MgOC_150_ adsorbed levofloxacin and some of β-lactams despite of small-sized molecules (Fig. 8). These drugs might be trapped by some residual hydroxyl groups within the MgOC_150_ material as well as pyocyanin. In contrast, the adsorption capability for amikacin, fosfomycin, and colistin was low (Fig. 8). For this reason, MgOC_150_ did not interfere with activities of amikacin, fosfomycin, and colistin (Table 5). Fosfomycin and colistin are well known as last resort agents in the treatment of MDRP strains (Michalopoulos et al., 2011, Montero et al., 2009). We suggest that MgOC_150_ may offer a benefit by assisting fosfomycin and colistin therapy.

Since MgOC_150_ is currently used for only industrial purposes, it is important to note that its safety must be carefully validated for medical applications, although this study provided evidence that MgOC_150_ does not impair human intestinal epithelial cells (Fig. 2B). Some extensive studies are necessary to be addressed whether MgOC_150_ adsorb any other molecules including beneficial compounds and fully understand the impact on host. However, microporous carbon adsorbents may open the door to the development of novel antimicrobial therapy.

## Author Contributions

H. Hirakawa, A. Kimura, K. Tanimoto, and H. Tomita designed the research, and wrote the manuscript. H. Hirakawa, A. Kimura, and H. Tomita analyzed the data. H. Hirakawa, A. Kimura, A. Takita and S. Chihara, K. Tanimoto performed the research.

## Funding

This research was funded by the Japan Society for the Promotion of Science (JSPS) “Grant-in-Aid for Scientific Research (C)” (Grant No. 19K07533 to H.H. and 21K06859 to A.K.) and “Grant-in-Aid for Scientific Research (B)” (Grant No. 22H02864 to H.H. and A.K.), the Takeda Science Foundation (H. H.), the Ohyama Health Foundation (H. H.), Japan Agency Research and development [AMED] (Grant No. 22fk0108604h0901 and 22wm0225008h0202 to H.T.) and Health Labour Sciences Research Grant (Grant No. 21KA1004 to H.T.).

## Declaration of Competing Interest

The authors declare that they have no known competing financial interests or personal relationships that could have appeared to influence the work reported in this paper.
